# Encephalomyelopathy associated with HTLV-I a primary disease or coexisting with multiple sclerosis?

**DOI:** 10.1590/S1980-57642013DN74000014

**Published:** 2013

**Authors:** Ana Paula Silva Champs, Valéria Maria de Azeredo Passos, Sandhi Maria Barreto, Paulo Caramelli, Carla Meirelles de Melo, Guilherme Carvalho, Miriam Melo Menezes, João Gabriel Ramos Ribas

**Affiliations:** 1 Department of Clinical Medicine, Medical School, Universidade Federal de Minas Gerais, Belo Horizonte MG, Brazil.; 2 Hospital Sarah Belo Horizonte, Belo Horizonte MG, Brazil.; 3 Post-Graduate Program in Adult Health Sciences, Medical School, Universidade Federal de Minas Gerais, Belo Horizonte MG, Brazil.; 4 Department of Preventive and Social Medicine, Medical School, UFMG, Belo Horizonte MG, Brazil.

**Keywords:** HTLV -I, tropical spastic paraparesis, multiple sclerosis, cognition disorder, magnetic resonance imaging

## Abstract

HTLV-I-associated myelopathy (HAM/TSP) is the most common neurological manifestation of HTLV-I, causing progressive weakness, sensory disturbance, and sphincter dysfunction. Although motor disorders have been well described, few studies have associated cognitive disorders and HTLV-I infection. In areas endemic for HTLV-I infection, the differential diagnosis between HAM/TSP and other myelopathy etiologies can be difficult, particularly if the patient has signs and symptoms of brain involvement, since seropositive HTLV-I patients can present other neurological diseases. Here, we report one case initially diagnosed as Multiple Sclerosis (MS) which, upon further investigation, was found to be HTLV-I seropositive.

## Abstract (pt)

A mielopatia associada ao HTLV–I (HAM/TSP) é a manifestação neurológica mais frequente do HTLV-I causando fraqueza progressiva, alterações de sensibilidade e disfunção esfincteriana. As alterações motoras são bem descritas, mas ainda são poucos os estudos que examinam a possibilidade de ocorrência de transtornos cognitivos na infecção pelo HTLV-I. Em áreas endêmicas para o HTLV-I, o diagnóstico diferencial com outras causas de mielopatias pode ser difícil, particularmente se o paciente tem sinais e sintomas de acometimento encefálico, já que a sorologia positiva para o HTLV-I pode ser detectada em pacientes com outras doenças neurológicas. Aqui relata-se o caso de uma paciente inicalmente diagnosticada com Esclerose Múltipla e que, na investigação posterior, foi encontrado soropositividade para HTLV-I.

## INTRODUCTION

HTLV (Human T cell lymphotropic virus) type I-associated myelopathy (HAM/TSP) occurs in 2%-3% of hosts, predominantly in females in their forties and fifties. Its onset is insidious and progression is slow. Gait disorders, weakness and lower limb stiffness are the outcome of a gradual decrease in muscle strength and spasticity in the affected myotomes. There is gradual disability, requiring walking-aids (canes and walkers) and ultimately may lead to use of a wheelchair. Discrepancy in the average time described for this development, from a few months to several decades, is explained by the difficulty in inferring the precise infection time upon serum diagnosis.^1^ Symptoms of bladder-bowel and sexual dysfunction disorders may be the patient's initial complaints, with bladder urge incontinence and intestinal constipation, as well as erectile dysfunction and lack of ejaculation in the male population. On neurological examination, signs suggestive of an upper motor neuron lesion can be seen, such as spasticity in the lower extremities, patellar and Achilles hyperreflexia and the presence of a Babinski reflex. It is important to emphasize the progressive nature of the disease, with no description of remissions.^1^

In areas endemic for HTLV-I infection, the differential diagnosis between HAM/TSP and other etiologies may be difficult, particularly if the patient has signs and symptoms of brain involvement, since HTLV-I antibodies may be detected in patients with other neurological diseases. Primary progressive multiple sclerosis (MS) may be particularly challenging because both conditions have inflammatory and immune-mediated behavior and are characterized by slowly progressive spastic paraparesis.

MS is distinguished by the presence of demyelination plaques and axonal loss in the brain and spinal cord, which can lead to development of various motor, sensory, sphincter, visual and cognitive signs and symptoms, depending on the location of the lesions. It can manifest in two ways: in outbreaks followed by remission, with transient signs and symptoms, occurring more often in young adults, or in a slow and progressive form developing neurological signs and symptoms without remission, more commonly beyond 40 years of age.^2^ This latter form, called primary progressive MS, has clinical features similar to HAM/TSP.

Ogata et al.^3^ suggested that brain magnetic resonance imaging (MRI) findings observed in HAM/TSP patients may be indistinguishable from those observed in MS patients. However, other authors suggest that patients with MS present a larger number of lesions^4,5^ that can be differentiated by location and size. They further suggest that brain MRI findings in MS show plaque and/or nodular lesions predominating in the periventricular white matter and the pericallous/septal area. According to Howard et al.,^5^ having a lesion of at least 6 mm in the supratentorial brain and an infratentorial lesion greater than 3 mm, large periventricular lesions, and T2-hyperintensity changes on cervical spinal cord MRI are more characteristic findings for MS than HAM/TSP.

HTLV-I cognitive disorders have been investigated following some case reports describing MRI brain abnormalities in patients with HAM/TSP. However, case reports and case series are not suitable research designs to demonstrate the association between cognitive impairment and HAM/TSP. On the other hand, cognitive impairment in MS is well described in scientific literature, particularly with regard to changes in executive functions and memory,^6^ which prevail in about 50% of patients. Such cognitive decline is usually found from the disease's early stages^6^ and can be the first neurological manifestation, mainly in progressive forms. For most MS patients, cognitive impairment represents the inability to function socially, occupationally and educationally.^6^

This report presents a patient with progressive paraparesis and cognitive changes referred for assessment of a potential diagnosis of MS, whose preliminary examination showed positive serology for HTLV-I.

## CASE REPORT

A 61-year-old, married, Caucasian woman with four years of schooling came to the appointment with her husband. Her husband completed the necessary information on the patient's medical history. She was admitted with a 10-year progressive gait disturbance associated with urinary incontinence. Six years before admission she presented increased leg weakness and became wheelchair-bound. She complained of recent memory changes and apathy starting a year earlier, but denied dysphagia, dysarthria or visual problems. In the past year she had also become somewhat reliant in her day-to-day activities, and needed help to bathe and dress.

She stopped doing some housework, and began just helping to make meals at home. Her husband reported being concerned about the patient, and felt she was a bit confused: for example, she could not visit people unless accompanied by a family member, as she would not know how to get back home. Currently, she is unable to use the phone or take her medications unassisted. Her husband had always been in charge of shopping and household finances and stated that the patient had never been able to handle money.She had never smoked or drunk, had no history of diabetes mellitus or arterial hypertension and had no family history of cognitive decline. She had a history of thyroidectomy in 1975, with levothyroxine replacement and regular assessment of thyroid function. She has been in use of 25 mg of amitriptyline for the last four years.

On admission, the patient was conscious, alert, anxious, talkative and insecure. Although the patient was anxious and insecure, the psychiatric evaluation did not reveal any specific disorder. Neurologic examination showed preservation in superficial tactile and pain sensitivity and spastic paraplegia with pyramidal signs.

Neurophysiological assessment by examining motor-evoked potential suggested a conduction defect in lower and upper limbs. Somatosensory-evoked potential tests and visual-evoked potential tests showed no changes.

The neuropsychological assessment showed a generalized poor performance on the tests (Table 1).

**Table 1:** Results of patient's neuropsychological assessment.

Neuropsychological Tests	Results
Mattis Dementia Rating Scale^7^	Attention: 29/37; Initiative and Perseverance: 28/37; Construction: 2/6; Conceptualization: 18/39; Memory:17/25. Total: 94.
Digit span in direct and inverse order^8^	Normal order 4 and reverse order 2
Verbal semantic^9^ and phonemic fluency^10^	Semantic: 10 animals; Phonemic: 11 of F;A;S
Trail making test^11^	Part A: 342 seconds; Part B: incapable
RAVLT^12^	A1:4; ITR (A6/A5): 1; ESQ (A7/A6): 1,4; REC: 7; A1-A5 = 27
Clock drawing^13^	Shulman score: 4
Stroop test^14^	Victoria version of StroopPhase I: 34", error free; Phase II: 38" error free; Phase III; 42", error free.

Blood examination was normal except for the presence of HTLV-I antibodies in serum (ELISA and Western blot). The Real-time PCR of DNA involved extraction from serum cells and proviral load was measured, showing 1027 HTLV-1 copies per 100,000 cells. The analysis of cerebrospinal fluid (CSF) showed a clear, colorless appearance, 12 cells/mm^3^, predominance of lymphocytes, glucose equal to 57 mg/dl and protein equal to 47mg/dl. Schistosomiasis, HIV and syphilis (VDRL) serum exams were all negative. The anti-HTLV antibody index for HTLV-I by ELISA was positive, as was the Real-time PCR of DNA measured proviral load of 12,905/100,000 cells. Elevated IgA, IgM and IgG, with an increased intrathecal IgG synthesis rate (1.51 mg/dl), was also observed. LCR protein electrophoresis revealed a polyclonal immunoglobulin increase and presence of oligoclonal IgG bands. Folic acid and vitamin B12 showed normal levels and investigation for vasculitis and other auto-immune diseases were negative (anticardiolipin, lupus anticoagulant, ANA, and ANCA all tested normal).

**Figure 1:**
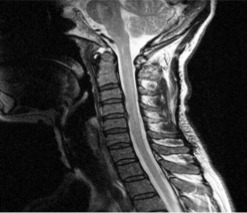
Cervical spine Magnetic Resonance Image of a flat sagittal spine medulla weighted at T^2^ (TR 3000 Te 111.8) on July 12, 2011 showing ill-defined areas of hyperintensity.

**Figures 2 and 3:**
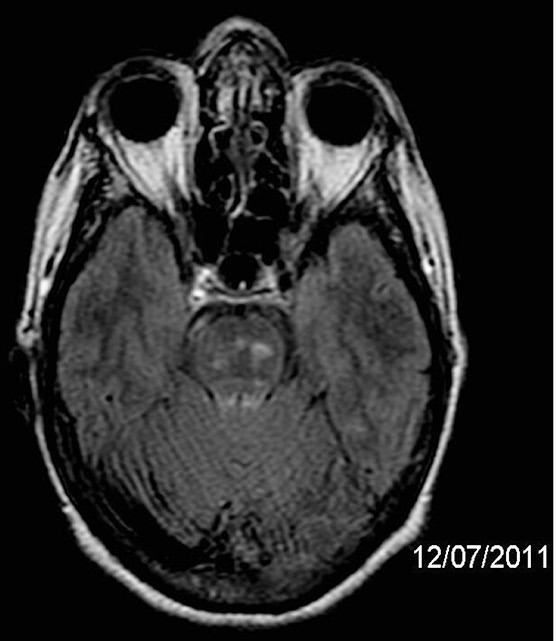

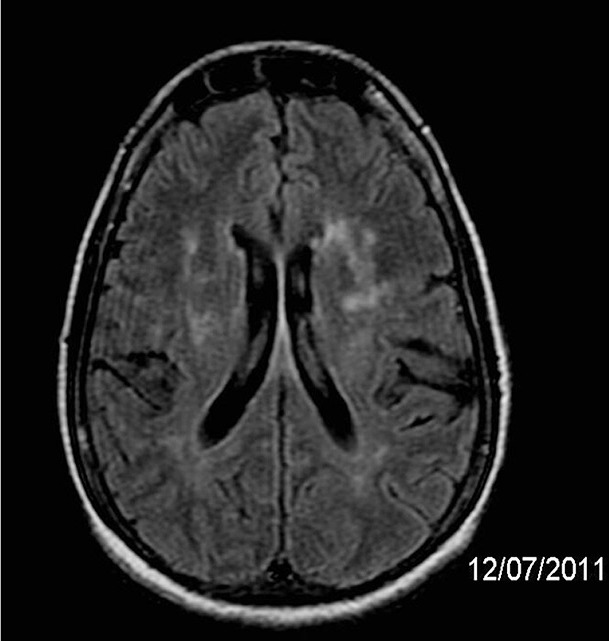
Brain Nuclear Magnetic Resonance Images of the axial planes using FLAIR (TR 9002.2, TE 152.2 TI 2200ms), on July 12, 2011 showing supra and infratentorial involvement.

MRI of the spine showed hyperintense lesion (T2-weighted sequences) in the medulla oblongata and cervical and thoracic spinal cord (Figure 1). MRI of the brain showed infratentorial and supratentorial lesions (Figures 2 and 3), characterized by hyperintense white matter lesions, some of which were confluent, in the upper portion of the lentiform nuclei, in the white matter of the radiated crowns, semioval, periventricular and subcortical centers in the brain hemispheres, pons, but no pericallous involvement. On proton spectroscopy, a slight increase in myo-inositol was noted in the signal change area of the left periventricular white matter.

**Ethical issues.** The patient and her husband authorized scientific publication of the medical information as well as the additional tests and signed a free and informed consent form. This research was authorized by the Research Ethics Committees of Hospital Sarah and Universidade Federal de Minas Gerais.

## DISCUSSION

Differentiating HAM/TSP from MS brain involvement can be difficult, particularly in endemic regions such as Brazil. Although the exact number is not known, an estimated 15-20 million persons are HTLV-I seropositive worldwide.^15^ In Brazil, a HTLV I/II seropositivity prevalence of 1-14% was detected in blood donors.

Cases of HAM/TSP mimicking MS have been reported^5,16^ as has the detection of HTLV-I antibodies in serum and CSF of patients diagnosed with MS exposed to infection by this virus.^17^ Here, we report a patient whose clinical development favors the diagnosis of HAM/TSP with brain involvement over MS. Although the case shares symptoms with the primarily progressive form of MS, this diagnosis is unlikely. Both diseases show gradual evolution of spastic paraparesis and sphincter disorder, but the development of brain involvement differs. The patient presented late generalized cognitive impairment, after many years of motor disorders. MS cognitive impairment is characterized mainly by decreasing speed of thought, impaired attention and memory decline which can be observed from the outset of evolution, sometimes as isolated neurological manifestations.^6^ HTLV cognitive impairment involves multiple domains, which is more compatible with this case.^18^ The patient also has no evidence of other secondary causes of cognitive deficit. Moreover, the absence of the visual impairment or alteration in visual-evoked potentials found in cases of MS^2^ supports the diagnosis of HAM/TSP rather than MS.

The presence of a high intrathecal synthesis of total IgG and oligoclonal bands in CSF are compatible with either MS or HAM/TSP, indicating chronic inflammation of the central nervous system with demyelinization, involving a humoral and cell immune response.^19^ Previous studies have found oligoclonal bands and an intrathecal IgG synthesis increase in 82% of patients with HAM/TSP^19^ and also in patients with a chronic form of MS.^2^ The presence of HTLV-I antibodies (ELISA) in CSF may be positive in HAM/TSP or MS with coincident infection with HTLV-I.^17^ However, we detected a high intrathecal HTLV-I proviral load in the CSF (12,905 copies of HTLV-1 copies per 100,000 cells) which is a strong biomarker of HTM/TSP able to differentiate this disorder from MS. Previous studies showed that the proviral load was higher in CSF in the majority of HAM/TSP patients than in MS patients and was higher in HAM/TSP than HTLV-I carriers.^17^

A conflicting aspect of this patient's diagnosis is the imaging test because the patient had fulfilled all MRI criteria for MS.^2,5^ Nevertheless, the typical findings of demyelinating pericallosal lesions and the presence of Dawson's fingers lesions were not found.^2^ The change found on spectroscopy is unspecific and may occur in any process in which myelin is degraded, a result compatible with the two diseases. In HAM/TSP^3^ the brain MRI findings revealed a lower number of white matter lesions^4,5^ of at least 3 mm and nonspecific brain abnormalities on T2-weighted images.

Studies of rare diseases, generally in the form of case reports or case series, are inconclusive, but have the merit of offering new hypotheses. The association between brain alterations and HTLV infection is not yet clear, but brain involvement in this infection seems probable and warrants further investigation.

In conclusion, after analysis of this patient's medical symptoms and tests, some important criteria were considered to suggest HTLV-I-associated brain impairment. Recent cognitive impairment was observed in a middle-age woman who presented long-term progressive paraparesis and absence of visual complaints. Also contributing to this hypothesis were the high proviral load both in LCR and in peripheral blood, the absence of pericallous lesions on brain MRI and normal visual-evoked potential.
